# The Cardioprotective Mechanism of Phenylaminoethyl Selenides (PAESe) Against Doxorubicin-Induced Cardiotoxicity Involves Frataxin

**DOI:** 10.3389/fphar.2020.574656

**Published:** 2021-04-12

**Authors:** Xiaoyu Fu, Mathew Eggert, Sieun Yoo, Nikhil Patel, Juming Zhong, Ian Steinke, Manoj Govindarajulu, Emine Akyuz Turumtay, Shravanthi Mouli, Peter Panizzi, Ronald Beyers, Thomas Denney, Robert Arnold, Rajesh H. Amin

**Affiliations:** ^1^ Department of Drug, Discovery and Development, Harrison School of Pharmacy, Auburn University, Alabama, AL, United States; ^2^ Department of Anatomy, Physiology and Pharmacology, College of Veterinary Medicine, Auburn University, Auburn, AL, United States; ^3^ Department of Chemistry, Recep Tayyip Erdogan University, Rize, Turkey; ^4^ Department of Electrical and Computer Engineering, Auburn University, Auburn, AL, United States; ^5^ Auburn University M.R.I. Research Center, Auburn, AL, United States

**Keywords:** doxorubicin, phenylaminoethyl selenides, frataxin, cardiomyopathy, cardiotoxicity, glutathione, mitochondrial metabolism

## Abstract

Doxorubicin (DOX) is an anthracycline cancer chemotherapeutic that exhibits cumulative dose-limiting cardiotoxicity and limits its clinical utility. DOX treatment results in the development of morbid cardiac hypertrophy that progresses to congestive heart failure and death. Recent evidence suggests that during the development of DOX mediated cardiac hypertrophy, mitochondrial energetics are severely compromised, thus priming the cardiomyocyte for failure. To mitigate cumulative dose (5 mg/kg, QIW x 4 weeks with 2 weeks recovery) dependent DOX, mediated cardiac hypertrophy, we applied an orally active selenium based compound termed phenylaminoethyl selenides (PAESe) (QIW 10 mg/kg x 5) to our animal model and observed that PAESe attenuates DOX-mediated cardiac hypertrophy in athymic mice, as observed by MRI analysis. Mechanistically, we demonstrated that DOX impedes the stability of the iron-sulfur cluster biogenesis protein Frataxin (FXN) (0.5 fold), resulting in enhanced mitochondrial free iron accumulation (2.5 fold) and reduced aconitase activity (0.4 fold). Our findings further indicate that PAESe prevented the reduction of FXN levels and the ensuing elevation of mitochondrial free iron levels. PAESe has been shown to have anti-oxidative properties in part, by regeneration of glutathione levels. Therefore, we observed that PAESe can mitigate DOX mediated cardiac hypertrophy by enhancing glutathione activity (0.4 fold) and inhibiting ROS formation (1.8 fold). Lastly, we observed that DOX significantly reduced cellular respiration (basal (5%) and uncoupled (10%)) in H9C2 cardiomyoblasts and that PAESe protects against the DOX-mediated attenuation of cellular respiration. In conclusion, the current study determined the protective mechanism of PAESe against DOX mediated myocardial damage and that FXN is implicitly involved in DOX-mediated cardiotoxicity.

## Introduction

Doxorubicin (DOX) is an anthracycline cancer chemotherapeutic agent that is used widely for the treatment of multiples cancers, including prostate, breast, lung, bone, and leukemias (Kuerer et al., 1999; Khan and Denmeade, 2000; Neville-Webbe et al., 2005). However, DOX use is also associated with the development of drug resistance and several adverse effects (Cersosimo and Hong, 1986; Lipshultz et al., 1991; Solem et al., 1996; Tada et al., 2002), including cumulative dose-dependent cardiotoxicity that leads to cardiac failure (CF) and has been reported to be one of most significant challenges associated with its clinical efficacy (Arola et al., 2000). Therefore, reducing DOX-induced toxicity without dampening its anti-cancer activity is critical to optimizing its use and effectively treating different cancers.

It has been well established that energy starvation plays an important role in the mechanism underlying DOX-induced cardiac failure (Ashour et al., 2012). Altered mitochondrial bioenergetics has attracted attention among researchers due to its association with reduced ATP generation and heart failure (Rajamanickam et al., 1979; Wang et al., 1999; Tokarska-Schlattner et al., 2006; Berthiaume and Wallace, 2007; Xu et al., 2020). For example, cardiac hypertrophy is associated with changes in mitochondrial morphology and numbers (Rajamanickam et al., 1979; Ong and Hausenloy, 2010). Furthermore, studies demonstrated that DOX-induced cardiomyopathy and the eventual cardiotoxicity was caused by mitochondrial iron accumulation (Kwok and Richardson, 2004; Mouli et al., 2015). Frataxin is a mitochondrial iron-sulfur cluster biogenesis protein and plays an important role in regulating mitochondrial energetics and cellular metabolism (Koutnikova et al., 1997). Patient’s with Friedreich’s Ataxia (FRDA) display an autosomal recessive mutation in the frataxin gene on chromosome nine and display symptoms such as ataxia, diabetes, limb failure, and severe cardiomyopathy (Durr et al., 1996; Campuzano et al., 1997). Previous reports from our lab have determined that DOX-induced cardiotoxicity was associated with attenuation of FXN. (Mouli et al., 2015). Furthermore, overexpression of FXN offered significant cardioprotection against DOX exposure (Mouli et al., 2015).

In the current study, we explored the novel application of a selenium-containing compound, phenylaminoethyl selenides (PAESe), as a potential therapeutic agent for reducing DOX-induced cardiotoxicity. Previous reports have shown that PAESe has antihypertensive and antioxidant activity (Herman et al., 1988; Pollock et al., 1988; Kang et al., 2011). Glutathione peroxidase, a cellular reductant, and well-known glutathione (GSH) replenisher was found to be directly involved and required in the redox cycling of PAESe antioxidant activity (De Silva et al., 2004; Cowan et al., 2011). Recently, we demonstrated that PAESe reduced DOX-induced cardiotoxicity in athymic mice with human prostate cancer xenografts by increasing cell viability and reducing reactive oxidant species (ROS) generation without diminishing the antitumor activity of DOX treatments (Kang et al., 2011; Cowan et al., 2011).

Based on a mechanistic understanding of Frataxin and the descriptive observations related to PAESe’s cardio-protective activity, we hypothesize that PAESe protects against DOX-induced cardiotoxicity through stabilizing mitochondrial bioenergetic metabolism and mitigating the attenuation of frataxin expression through antioxidant mechanisms by recycling GSH. Here we describe the molecular basis for the observed cardioprotective activity of PAESe against DOX-induced cardiotoxicity.

## Methods and Materials

### Animals

Juvenile NCr athymic (*nu/nu*) mice were obtained from Taconic (Tarrytown, NY) and maintained according to an approved Institutional Animal Care Use Committee protocol at Auburn University. Mice were housed in a controlled environment (23°C; 12:12-h light-dark cycle) with *ad libitum* access to water and standard chow diet. Juvenile (6–8 week old) mice were randomly divided into four groups, saline (control), DOX, DOX + PAESe, PAESe. Mice received weekly tail vein injections of saline, DOX (QIW 5 mg/kg in saline x 5) for a cumulative dose of 25 mg/kg of DOX, PAESe in saline (10 mg/kg), or DOX + PAESe (QIW 10 mg/kg x 5) over four weeks followed by two weeks of recovery with no treatment. At two weeks after the last dose, hearts were surgically removed immediately after exsanguination, snap-frozen in liquid nitrogen, and stored at −80°C for further use.

### MRI Measurements of Cardiac Parameters

Magnetic resonance imaging (MRI) was completed at the AU-MRI Research Center. Cardiac volumetric functional parameters for left ventricular (LV) end-diastolic (E.D.) volume, end-systolic (ES) volume, ejection fraction (EF), ejection volume (EV), and LV mass were calculated using Matlab (Mathworks, Natick, MA) and bull’s eye graphing. Wall thickness was calculated as the radial distance between the endo- and epicardial contours and wall thickening as percent change from the end-diastolic (ED) wall thickness to end-systolic (ES) wall thickness. Both endo- and epicardial contours were traced on the short-axis (SA), and images that were acquired were at the E.D. and E.S. phases. The values attained from the areas defined by these contours included the associated per-slice E.D. volume (EDV), E.S. volume (ESV), LV ejection fraction (LVEF), LV ejection volume (LVEV), and thus allowing the LV mass to be calculated; where LV mass = LV volume * 1.055 g/cm^3^. The sum of the individual S.A. slice parameters provided whole-LV volumetric parameters.

### Cell Cultures

Rat-derived embryonic ventricular cardiomyoblast (H9C2) cells were purchased from ATCC (CRL-1446) and cultured according to the manufactures recommendations. In brief, cells were grown in DMEM supplemented with 10% (v/v) fetal bovine serum and 1% (v/v) Pen-Strep. Before drug treatment, cells were counted and plated with equivalent numbers of cells (density) based upon plate size into several 100 or 150 mm^2^ plastic cell culture plates for drug treatment.

### Drug Treatment *In Vitro*

DOX was purchased from Sigma-Aldrich (St. Louis, MO), and phenylaminoethyl selenide (PAESe) was synthesized and provided by Dr. Sheldon May (Georgia Tech). DOX and PAESe were reconstituted in DMSO. Cells were treated with complete DMEM media containing the desired concentration of drugs for 24 h. In this study, cells were treated with or without DOX (10 µm) for 24 h, followed by incubation with or without PAESe (1 μM) for an additional 24 h.

### Western Immunoblot

Heart tissue lysates were homogenized using a polytron tissue disruptor in cell lysis buffer (Cell Signaling Technologies cat. #9803) with protease cocktail inhibitor (Thermo Sci. cat. #87786). Proteins were extracted from H9C2 cardiomyoblasts and heart tissue with cell lysis buffer and supplemented with protease cocktail inhibitor. Proteins were standardized using a Nanodrop (Thermo Scientific). Changes in FXN levels were measured by western analysis as described previously using FXN (1:1,000, SantaCruz, cat. #25830) antibody (Mouli et al., 2015; Nanayakkara et al., 2015). Briefly, protein lysates were denatured, resolved and blotted on a nitrocellulose membrane. The blots were stained with primary antibodies, FXN (1:1,000, Santa Cruz, cat. #25830) and α-tubulin (1:2,000, DSHBY, cat. #12G10), washed with Tris-buffered saline-Tween and incubated with a secondary - horseradish peroxidase-conjugated antibody (1:2,000, Rockland) for 1 h. Blots were visualized with chemiluminescence reagent (Millipore) and imaged on a Bio-Rad gel dock system. Protein bands were analyzed using Quantity One software (BioRad) or ImageJ software and standardized to α-tubulin.

### Gene Expression

Changes in markers (gene expression) associated with ventricular size enlargement were measured by real-time PCR analysis. Total R.N.A. was extracted from animal tissues and cultured H9C2 cardiomyocytes with or without DOX using Trizol (Invitrogen) following the recommended protocol from Invitrogen. Briefly, heart tissues were crushed in liquid nitrogen with mortar and pestle into a fine powder. Trizol was then added to each sample and combined with 0.1 ml chloroform into each tube. Partition containing R.N.A. was isolated by centrifugation (12,000 g for 15 min at 4°C). The upper aqueous phase containing R.N.A. was isolated and combined with isopropanol, centrifuged again at 12,000 g for 10 min at 4°C. R.N.A. pellet was washed with 75% ethanol, and then centrifuge at 7,500 g for 5 min at 4°C. R.N.A. pellets were then air-dried and reconstituted into RNAse free water. cDNA was synthesized from R.N.A. using a first strand OneScript cDNA synthesis kit (A.B.M.).

Markers of cardiac hypertrophy were detected by qPCR analysis using a BrightGreen 2X qPCR MasterMix (A.B.M.). Sequences of probes for β-MHC are as follows: Forward 5′-AAG GAG GAG TTT GGG CGA GTC AAA-3′ and reverse 5′-TGC ATC CGC CAA GTT GTC TTG TTC-3’. Sequences of probes for A.N.P. are as follows: Forward 5′-AGA CAG CAA ACA TCA GAT CGT GCC-3′ and reverse 5′-ATC TGT GTT GGA CAC CGC ACT GTA-3’. Sequences of probes for β-Actin are as follows: Forward 5′-TTG CTG ACA GGA TGC AGA AGG AGA-3′ and reverse 5′-ACT CCT GCT TGC TGA TCC ACA TCT-3’. Data analysis was conducted using ΔΔCt method (Zhang et al., 2013; Pabinger et al., 2014).

### Aconitase Assay

Aconitase activity was measured using Aconitase Assay Kit (Cayman chemicals, cat. #705502). Mechanistically, aconitase catalyzes the isomerization of citrate to isocitrate, which is then converted to α-ketoglutarate by isocitrate dehydrogenase. Cells were grown in 150 mm^2^ plates and treated with or without DOX (10 µM) for 24 h, followed by incubation of PAESe (1 μM). Aconitase enzyme activity was monitored from isolated mitochondria and based on assay protocol by measuring the change in absorbance at 340 nm that is associated with the formation of NADPH using a Cytation 5 (BioTek) multimodal imaging reader. Values were standardized by protein concentrations, as measured by Nanodrop (Thermo).

### Cardiac Troponin Assay (cTnI)

Cardiac troponin-I (cTnI) is a component of the troponin complex that regulates cardiac muscle contraction. After cardiac injury, cTnI is released into the circulation. The current ELISA measures troponin expressed specifically in cardiomyocytes and therefore it is an excellent biomarker of cardiac injury. ELISA for mouse cTnI was purchased from Life Diagnostics (Cat # CTNI-1-HS). Briefly, cTnI was measured from serum samples (100 μL). Values were determined from samples by the endpoint method, where plates are measured at 450 nm after 5 min at room temperature using a Cytation five multimodal imaging reader. Concentrations of cTnI from measured samples were based upon given concentration curve of mouse cTnI from the ELISA kit. Final values were represented as per ml of blood.

### Glutathione Assay

Glutathione peroxidase activity was measured using a Glutathione Peroxidase Assay Kit (Cayman Chemical). Cells were grown in 150 mm^2^ plates and treated with or without DOX (10 µM) for 24 h, followed by incubation of PAESe (1 μM). Briefly, heart tissue samples from mice were washed with PBS and then snap-frozen and ground to a powder with liquid nitrogen using a mortar and pestle (as per instructions form the kit). Tissue and cell pellets were reconstituted in MES buffer (supplied in kit). Values were standardized by protein concentrations, as determined by Nanodrop (Thermo). Glutathione peroxidase activity from samples was determined by the endpoint method, where plates are read at 405–414 nm after 25 min at room temperature using a Cytation five multimodal imaging reader.

### Mitochondrial Respiration

Cellular respiration was measured as previously described (Koves et al., 2005). Following DOX (10 µM) with or without PAESe (1 µM) treatment, H9C2 cells were washed in PBS, trypsinized and centrifuged at 800 g for 3 min. Cellular particulate pellets were then washed and resuspended in 400 μL PBS containing 10 mM glucose, 10 mM HEPES, 0.2% B.S.A. and pH 7.45 and maintained at 37°C. After that, 300 μL of glucose supplemented PBS was added to the oximeter chamber (Hansatech Instruments, Pentney, United Kingdom) and equilibrated for approximately 1 min after which, 350 μL of the cell suspension solution was added. The basal respiration rate was then recorded for approximately 5 min. Subsequently, 6 μM FCCP (OXPHOS uncoupler) was added, and respiration was monitored for an additional 2 min. Basal (coupled) and maximal (uncoupled) respiration rates were normalized to protein concentrations from cells (50 µL of aliquot) remaining in supplemented PBS.

### Mitochondrial Membrane Potential Assay

The mitochondrial membrane potential was measured utilizing tetramethylrhodamine ethyl ester, TMRE (Biotium, cat. #70016), a positively charged red-orange dye that accumulates in active mitochondria. H9C2 cells treated with or without DOX (10 µM) plus or minus PAESe (1 µM) were plated in a 12 well plate, and mitochondrial membrane potential was determined. Depolarized or inactive mitochondria have reduced membrane potential and therefore fail to sequester TMRE. The cells were stained according to the manufacture’s protocol, and the signals were measured (ex/em at 548/575 nm) using a Cytation five multimodal imaging reader.

### ATP Assay

The ATP levels were quantified based on a protocol using the ATPlite luminescence ATP detection assay system (PerkinElmer, no. 6016941). H9C2 cells were plated based upon a standardized cell number using a hemocytometer. Cells were then treated with increasing concentrations of DOX (1–50 µM) with or without PAESe (1 µM). Briefly, the bioluminescence represents the reaction between D-luciferin and ATP and was proportional to the ATP concentrations. The luminescence was measured using Glomax luminometer (Promega). Values were standardized to total protein concentrations per well.

### Mitochondrial Iron Detection: Ferrozine Colorimetric Assay

Iron detection was performed using a Ferrozine colorimetric assay, which is based upon previously published technique for detection and quantification of free iron by forming a colorimetric complex (Riemer et al., 2004; Nanayakkara et al., 2015). Cultured H9C2 cardiomyocytes were treated with or without DOX (10 µM) for 24 h, followed by incubation with or without PAESe (1 μM). Mitochondrial and cytosolic fractions were isolated from ventricular lysate and H9C2 cell lines and lyzed with NaOH lysis buffer for 45 min. Cellular fractions were then incubated with H.C.L./KMnO_4_ for 2 h at 60°C, which enhanced the detection of iron in the samples. Absorbance was read at 550 nm and standardized to total protein concentrations.

### ROS Measurement

Mitochondrial reactive oxygen species (ROS) was measured using mitochondrial specific dihydrorhodamine (DHR) assay (Biotium). DHR is a ROS indicator that accumulates in the mitochondria and is oxidized to form the charged cationic rhodamine 123, where it exhibits a green fluorescence (505/534 nm). The cells were seeded in a 24-well plate and stained according to the manufacturer's protocol. Briefly, cells were treated with or without DOX (10 µM) for 24 h, followed by incubation of PAESe (1 μM). Fluorescence intensity was detected using a Cytation five multimodal image reader, and analysis was accomplished using Gen5 software and ImageJ software per treatment from four independent sets of experiments. The total fluorescence values were obtained by comparisons of densitometric values to the total area.

### Statistical Analysis

Statistical comparisons between groups were determined using paired Student’s t-test, and one-way ANOVA was applied to analyze for multiple groups. Data were reported as means ± S.E.M. A *p*-value ≤0.05 was considered to be a significant difference between groups. All experiments were accomplished with a minimal of N = 3 replicates in three independent experiments.

## Results

### PAESe Mitigates from the Development of Ventricular Enlargement and Impaired Cardiac Performance (Figure 1)

**FIGURE 1:**
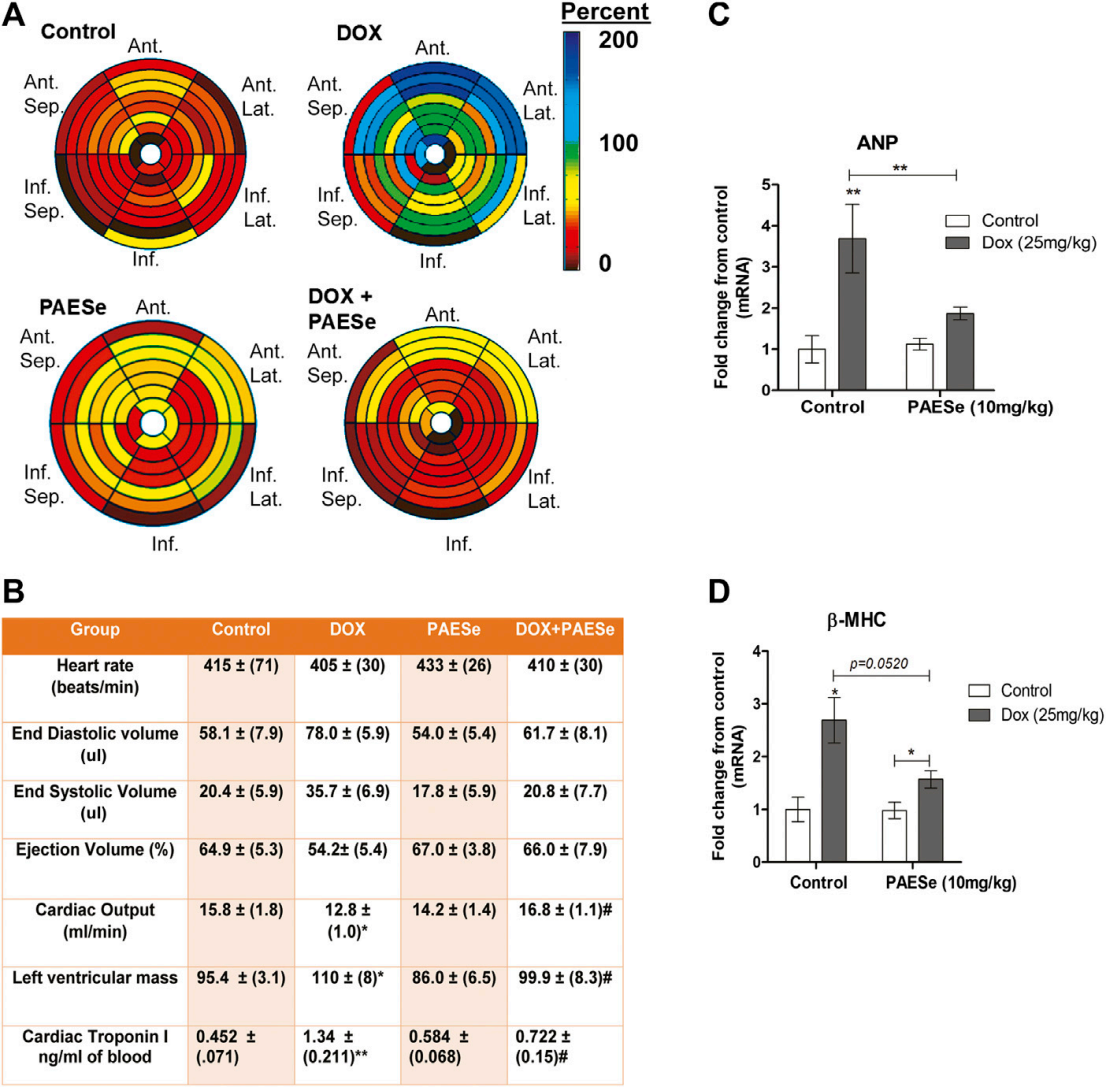
PAESe protects against DOX-induced cardiac hypertrophy. **(A,B)** PAESe reduces DOX mediated changes in cardiac architecture and performance in athymic mice as determined from MRI analysis. 16 segment model of the LV portion of the Bull’s eye plot from MRI scans show the anterio-lateral geometry of the left ventricle (LV) in DOX treated mice (Ant, Anterior; sep, septum; Lat, Lateral; Inf, Inferior). Further MRI the analysis revealed an increase in left ventricular mass index, the effects of DOX on cardiac performance revealed a reduced end-diastolic volume and end-systolic volume, as well as reduced heart rate, ejection volume and overall cardiac output. Measurements of cardiac troponin (cTnI) from serum samples revealed the presences of cardiotoxicity and that PAEse offered cardioprotection against Dox mediated cardiotoxicity. **(C,D)** Measurements of changes in markers for cardiac hypertrophy (ANP and β-MHC) as measured by qRT-PCR analysis in athymic mice treated with Doxorubicin (DOX) (5 mg/kg, one dose per week for 5 weeks, 2 weeks recovery). Values were based on ΔΔct values, which were standardized to β-actin expression. Statistical differences were determined by a student t-test, and comparisons were analyzed based on SEM where n = 4 animals per group. Values are represented as mean ± S.E.M. and were analyzed by student t-test where N = 4 animals per group. *; *p* < 0.05. **;*p* < 0.001

**FIGURE 2:**
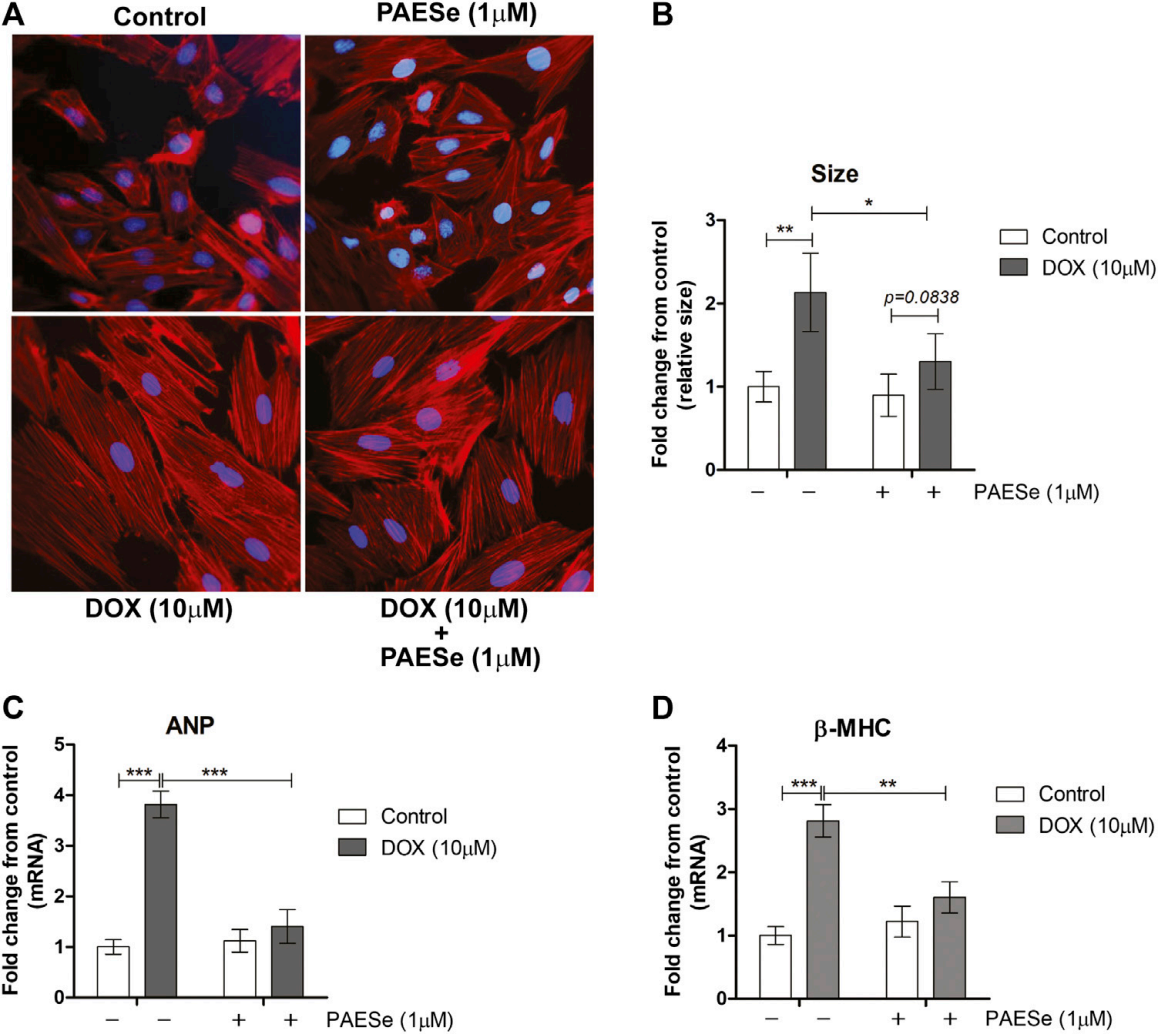
PAESe protects against DOX-induced cardiac hypertrophy in H9C2 cardiomyoblasts. **(A)** Representative phalloidin fluorescence imaging displays protective effects by PAESe (1 µM) against DOX (10 µM) mediated hypertrophy in H9C2 cell as demonstrated by fold change in size by treatment. **(B)** Graphical measurement of morphometric measurements from panel **A**. (**C**,**D**) ANP and β-MHC mRNA expression were evaluated by two-step qPCR analysis from cells treated with DOX (10 µM) with and without PAESe (1 µM) for 24 h. Data are represented as fold change from control and in the case for qPCR analysis standardized to β-actin. Indicated above are the mean ± S.E.M. of the replicates, N = 4 individual experiments, **; *p* < 0.001, ***; *p* < 0.0001, ###; *p* < 0.00001.

**FIGURE 3:**
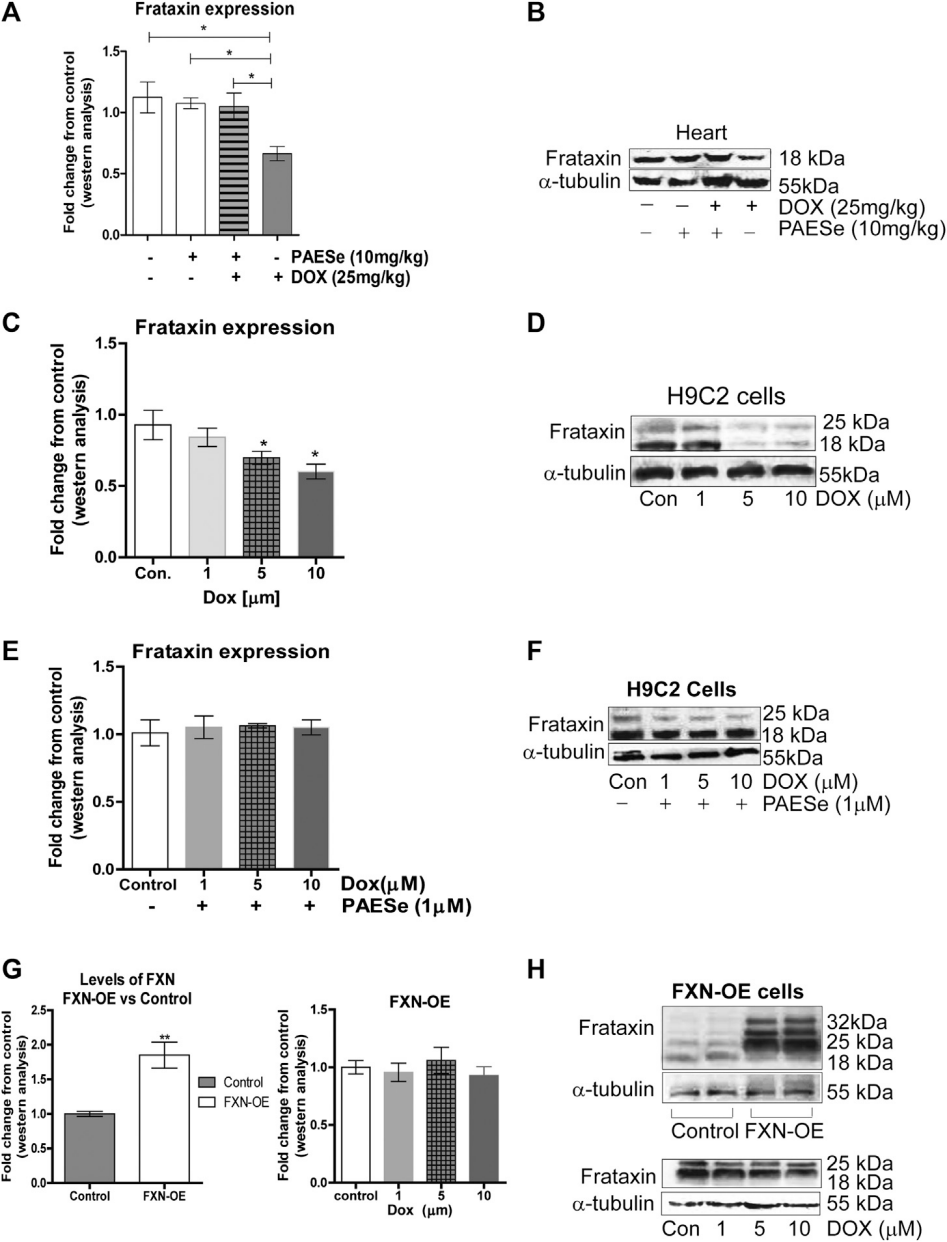
PAESe prevents DOX-mediated reduction of frataxin expression. **(A,B)** Western blot analysis of frataxin expression levels from heart tissue from cumulative dosing of DOX (25 mg/kg) ± PAESe (50 mg/kg/dose) as described in the methods section. Animals were administered drugs once a week (Q.I.W.), and the dosing stopped at the 4th weeks. (*p < 0.05; **p < 0.001, ***p < 0.0001; Mean ± SEM, n = 3x4) **(C,D)** Western blot on H9C2 and **(E–H)** Frataxin over expressing (OE) H9C2 cardiomyobalsts cell lines. Both H9C2 and Frataxin overexpressing cells were treated with drug for 24 h as control (media), DOX (1 μm, 5 μm, or 10 µm) with or without PAESe (1 µM). (*p < 0.05,**p < 0.001, ***p < 0.0001 vs Control; Mean ± SEM, N = 3).

Chronic administration of DOX at clinically relevant concentrations is known to induce cardiomyopathy, which eventually progresses the heart towards failure (Wang et al., 1998; Karagiannis et al., 2010). The impact of PAESe on chronic DOX (5 mg/kg, QIW x 4 weeks with 2 weeks recovery) mediated changes in cardiac architecture and performance as evaluated in mice using MRI. Following DOX treatment, MRI imaging and analysis revealed an increase in the left ventricular mass (1B) [t (6) = 3.450; *p* < 0.05]. Bull’s eye maps were used to illustrate changes in ventricular mass by computational analyses of the cardiac architecture. DOX treatment resulted in an enlargement in the anterolateral segment of the left ventricle by evaluation of changes associated with the left ventricular segmented regions (Figures 1A,B). Further MRI analysis measured significant changes in cardiac output in DOX treated mice when compared to control mice [t (10) = 2.914; *p* = 0.0155]. Further that PAESe protected against the reduced cardiac output mediated by DOX [t (10) = 2.691; *p* = 0.0227]. However, mice treated with DOX plus PAESe did not show enlargement in the anterolateral and lateral regions of the heart (Figures 1C,D). However, the addition of PAESe to DOX treatment appeared to protect against this observed increase (non-significant) in mean LV mass [t (6) = 1.787; *p* = 0.124]. Markers associated with cardiac hypertrophy including atrial natriuretic peptide (A.N.P.) (∼4 fold) [t (6) = 4.296; *p* = 0.005] and beta-myosin heavy chain (β-MHC) (∼3.5 fold) [t (6) = 2.418; *p* = 0.052] were also evaluated following DOX treatment when compared to control mice (Figures 1D,E). Together, these data reveal an increase in the size of the left ventricle from mice treated with DOX when compared to control mice. To further test that PAESe confers protection on cardiac performance in DOX treated mice, we observed by MRI analysis, reduced end-diastolic and end-systolic volumes, as well as reduced heart rate, ejection volume, and overall cardiac output (Figure 1B). These data together suggest that PAESe protects the heart against the chronic regimen of DOX that induces compensatory ventricular hypertrophy and potentially progresses to cardiac decompensation. However, DOX also induces cardiotoxicity and therfore we measured circulating levels of cardiac troponin. We observed a significant increase after 2 weeks recovery from cumulative dosing of DOX [t (10) = 3.954; *p* = 0.0027]. We also observed that PAESe reduced DOX mediated increase in cTnI levels in circulation [t (10) = 2.397; *p* = 0.0381]. In Figure 2, our *in vitro* findingsobserved that DOX (10 µM) induces an increase in H9C2 cardiomyoblast size [t (6) = 4.240; *p* = 0.0280]. Further that PAESe protected against DOX mediated increase in H9C2 cardiomyoblast size [t (8) = 1.974; *p* = 0.838] as measured by surface area (2A) and gene expression studies (Figures 3C,D) [ANP (t (8) = 12.68; *p* = 0.0001)], [MHCβ (t (8) = 3.405; *p* = 0.0093)].

### PAESe Mitigates DOX-Induced Cardiotoxicity by Increasing Frataxin Expression Levels (Figure 3)

In the current study, we observed a significant reduction in FXN protein expression in ventricles of hearts from DOX treated athymic mice when compared to our control-treated athymic mice (Figures 2A,B) [t (4) = 3.309; *p* = 0.0297]. Furthermore, that PAESe abrogated the deleterious effect of DOX on FXN expression (Figures 2A,B) [t (4) = 3.037; *p* = 0.385]. These findings were confirmed in H9C2 cells after exposure to 10 μM DOX, resulting in a significant decrease in both forms of FXN (mitochondrial (18 kDa) and cytosolic (24 and 32 kDa)) after 24 h (Figure 3B). However, PAESe was observed to preserve the reduction of FXN by increased concentrations of DOX (Figures 2C,D). To further understand the role of FXN in the cellular signaling events leading to cardiac hypertrophy, we constructed Frataxin overexpressing (FXN-OE) H9C2 cardiomyocyte cell lines which have been previously described (Nanayakkara et al., 2015). In the current study, we observed minimal reduction in response to DOX (10 µM) after 24 h (Figures 2E,F). Our results suggest that increasing DOX concentrations leads to reduced expression FXN. However, these findings were not replicated in our FXN-OE cells.

### DOX Mediated Reduction in Frataxin Attenuates Mitochondrial Iron Homeostasis (Figure 4)

**FIGURE 4:**
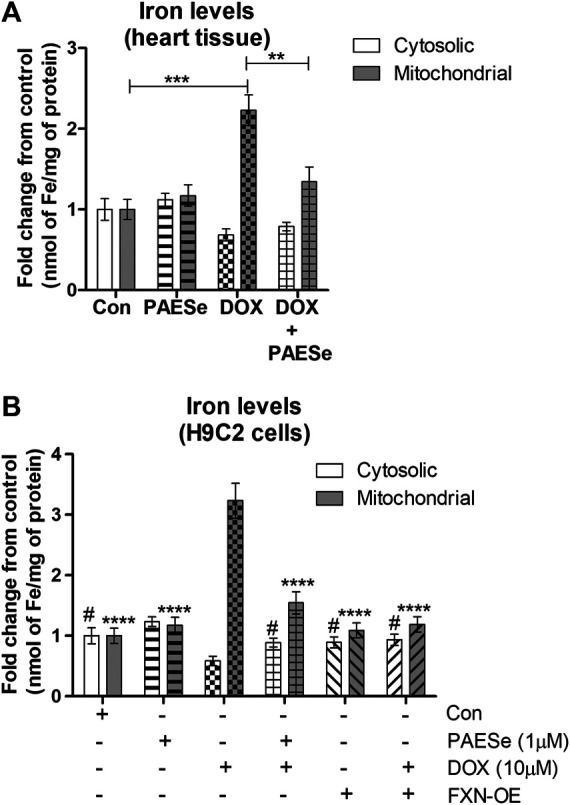
PAESe protects against DOX mediated mitochondrial iron accumulation. **(A)** Ferrozine assay from heart tissue. Mitochondrial and cytosolic fractions were partitioned from heart tissues collected from control and drug-treated nude mice that were imaged by MRI in Figure 1. Groups were divided as the following: Control (saline), DOX (25 mg/kg/Dose), PAESe (10 mg/kg/dose) and DOX + PAESe (25 mg/kg/dose+50 mg/kg/dose) as indicated in the methods section. Animals were administered drugs once a week (Q.I.W.), and the dosing stopped at the 4th week, followed by two weeks of recovery. (*p < 0.05,**p < 0.001, ***p < 0.0001; Mean ± SEM, n = 4 mice per group). Mitochondrial and cytosolic iron levels were measured by Ferrozine colorimetric assay. **(B)** Free iron (Fe^2+^/^3+^ was measured by a Ferrozine colorimetric assay based upon a previously published technique (Riemer et al., 2004), from mitochondrial and cytosolic H9C2 subcellular fractions. Cells were treated with DOX (10 µM) with or without PAESe (1 µM) for 24 h (*p < 0.05,**p < 0.001, ***p < 0.0001 vs Control; Mean ± SEM, N = 4).

FRDA fibroblasts and yeast mutants (ΔYFH1) display attenuated levels of FXN expression. As a result, both cells display elevated mitochondrial iron accumulation (Cavadini et al., 2000; Huang et al., 2009; Chen et al., 2013). Therefore, we postulated that reduced FXN expression by DOX alters the sub-cellular iron distribution and thereby leads to an increase in mitochondrial iron accumulation. Furthermore, PAESe inhibits the reduction of FXN, resulting in preventing the elevation of mitochondrial iron accumulation. Our DOX treated mice displayed elevated mitochondrial iron accumulation (∼2.2 fold increase) and conversely reduced cytosolic iron levels when compared to control mice, as demonstrated by results from our Ferrozine colorimetric iron assay (Figure 4A) [t (10) = 13.34; *p* = 0.0001]. Most importantly, we observed that the co-administration of PAESe with DOX in our animal studies prevented the accumulation of iron in the mitochondria [t (10) = 8.254; *p* = 0.0001]. To examine this at a cellular level, we observed that DOX-induced a significant increase in mitochondrial iron accumulation in H9C2 cardiomyoblasts (Figure 4B), [t (9) = 17.25; *p* = 0.0001]. Furthermore, we demonstrated that PAESe treatment inhibited DOX-mediated increases in iron accumulation [t (9) = 11.80; *p* = 0.0001]. These results support the hypothesis that PAESe protects against DOX-mediated cellular iron dyshomeostasis, and FXN expression is involved in the protective mechanism underlying PAESe’s role in preventing mitochondrial iron overload.

### PAESe Attenuates DOX Mediated ROS formation and Maintains Mitochondrial Membrane Potential (Figures 5, 6)

**FIGURE 5:**
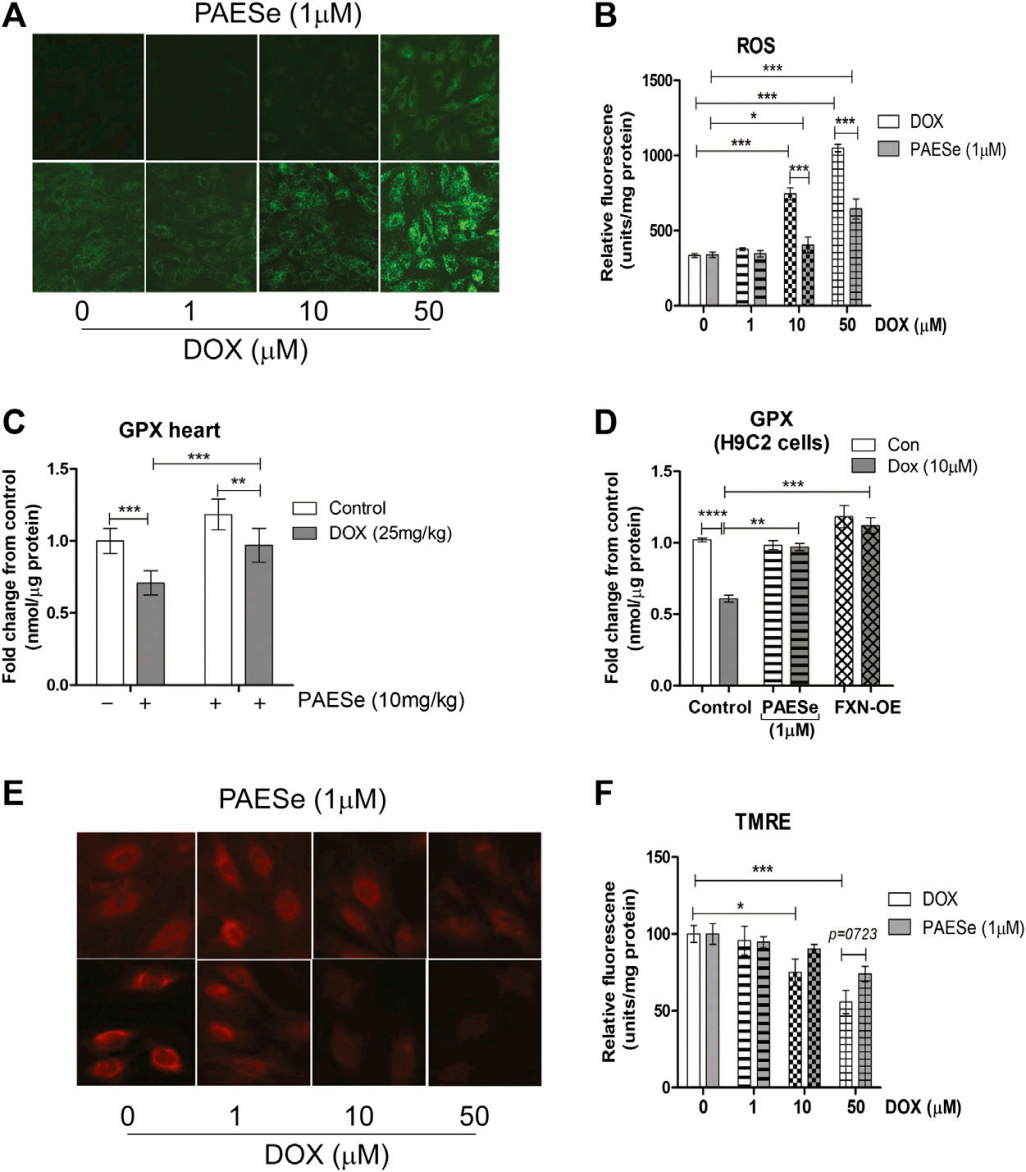
PAESe protects against DOX mediated mitochondrial ROS formation. **(A,B)** Fluorescence images of mitochondrial ROS formation (green) in increasing concentrations of DOX (0–50 µM) treated H9C2 cells with or without PAESe (1 µM) and graphical representation of ROS levels in H9C2 cardiomyoblasts. **(C)** Glutathione peroxidase activity, mitochondrial heart lysate from DOX treated athymic mice with and without PAESe (cumulative dosing of (DOX 25 mg/kg) ± PAESe (50 mg/kg/dose). **(D)** In addition, GPx activity was measured in isolated mitochondria from H9C2 cells treated with DOX (10 µM) or PAESe (1 µM) treated and non-treated H9C2 and FXN-OE cardiomyoblasts. **(E**,**F)** Mitochondrial membrane potential (Δψ_m_) determined by TMRE (2 μM) immunofluorescence in DOX treated (1–50 µM) H9C2 cells with and without PAESe (1 μM) for 24 h where N = 4 independent experiments and are represented as mean ± S.E.M. and analysis was accomplished by student t-test with post-hoc analysis for significances between groups and within groups based upon averages from the assays. *;*p* < 0.05 **; *p* < 0.001, ***; *p* < 0.0001.

Previous reports confirm that DOX, induced mitochondrial iron accumulation, enhances R.O.S. formation, and oxidative stress in cardiomyocytes (Mouli et al., 2015). Therefore, the maintenance of iron homeostasis in mitochondria is vital for cell survival. Based on our observations, that PAESe mediates mitochondrial iron homeostasis in the presence of DOX by preventing alterations in the expression of Frataxin. We observed that H9C2 cardiomyoblasts treated with increasing concentrations of DOX elevated mitochondrial ROS formation (Figures 5A,B) [t (10) = 63.46; *p* = 0.0001]. Furthermore, PAESe protected against the DOX-mediated increase in ROS formation [t (10) = 14; *p* = 0.0001]. To further explore this mechanism, we demonstrated that PAESe inhibited DOX-mediated reductions in glutathione peroxidase (GSHPx) activity in mice hearts (Figure 5C), [t (4) = 13.01; *p* = 0.0002] and H9C2 cardiomyoblasts [t (4) = 10.37; *p* = 0.0005]. Selenium is an vital element of selenocysteine, the active site of GSHPx (Ursini and Bindoli, 1987; Gladyshev et al., 1996). GSHPx is an antioxidant enzyme that reduces oxidant-induced DNA damage and lipid peroxidation. Further reduced levels of GSHPx is highly correlated with the initiation and progression of malignancy (Sundstrom et al., 1984; Ursini and Bindoli, 1987). The ability of PAESe to replenish the cellular anti-oxidative capacity with glutathione is to effectively improve mitochondrial iron homeostatic levels, which are in excess (Kang et al., 2011; Kang et al., 2015).

**FIGURE 6:**
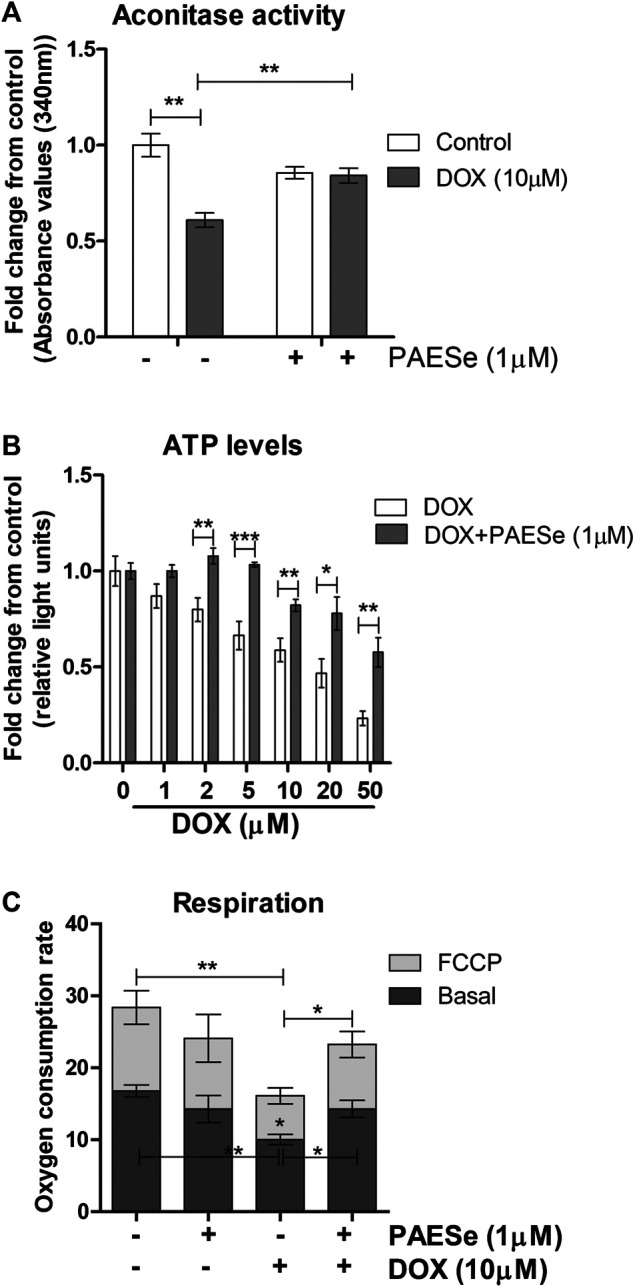
PAESe protects against DOX-mediated attenuation of mitochondrial bioenergetics. **(A)** H9C2 cells were treated as following; control (media), DOX (10 µm), PAESe (1 µM), and DOX (10 µM) + PAESe (1 µM). Mitochondrial isolations were extracted from cells for aconitase activity assay. Values were standardized to total protein concentrations, based upon on a standard curve of enzyme activity from the kit, and compared to control levels. **(B)** ATP levels were measured from H9C2 cardiomyoblasts treated with increasing concentrations of DOX (0–50 µM), for 24 h treated with and without PAESe (1 µm) (N = 4 independent experiments with three repetitions and values are based upon averages ± SEM. **(C)** Mitochondrial respiration (basal and uncoupled (FCCP)) in H9C2 cardiomyoblasts were measured following DOX (10 µm) with our without PAESe (1 µm) for 24 h. Values were normalized to total protein levels. The mitochondrial complex activity was decoupled by adding Trifluorocarbonylcyanide (FCCP; 6 µM), where N = 4 independent experiments. (*p < 0.05,**p < 0.001, ***p < 0.0001; Mean ± SEM, N = 4 independent experiments with N = 3 repetitions per group).

### PAESe Improves Mitochondrial Bioenergetics (Figure 6)

**FIGURE 7:**
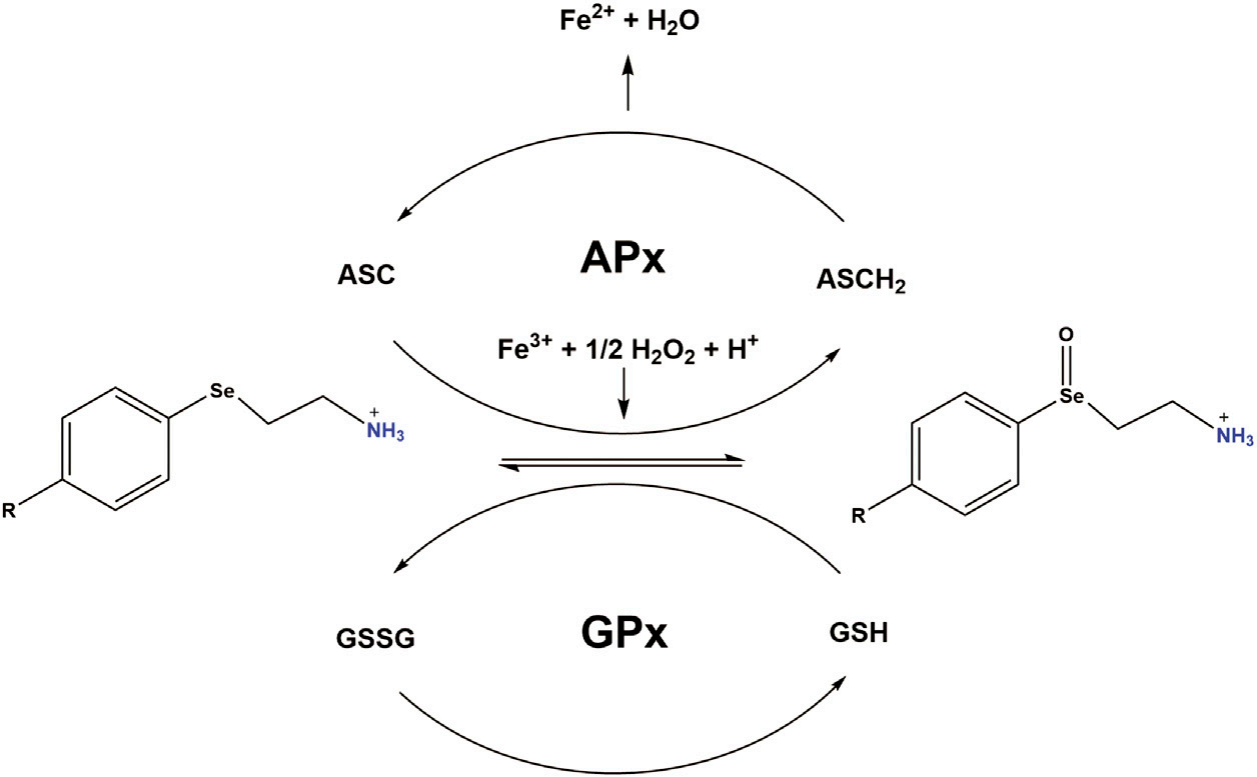
Working model: PAESe is oxidized to its selenoxide PAESe(O) form via dopamine-β-monooxygenase. Reduced Ascorbate (ASCH_2_) reduces PAESe(O) back to the reduced form generating oxidized ascorbate (ASC) in the process. ASC is then capable of reacting with PAESe and hydrogen peroxide to reduce Fe^3+^ back to Fe^2+^. Catalytic regeneration of PAESe is also enhanced via Glutathione Peroxidase (GPx) reduction with glutathione (GSH), yielding glutathione disulfide (GSSG). Regeneration of PAESe enables mitochondrial ROS formation to be ameliorated at a much faster rate compared to normal physiological conditions, allowing for potent free radical scavenging and enhanced mitochondrial function. The working model is adapted from a figure by May et al. (May and Pollock, 1998).

Previous reports have observed reduced mitochondrial respiration in FXN lacking cellular models, including fibroblasts isolated from FRDA patients (Bradley et al., 2000; Vorgerd et al., 2000; Mühlenhoff et al., 2002). Our DOX treated H9C2 cardiomyoblasts displayed reduced FXN expression levels and increased mitochondrial ROS formation. We then tested that PAESe ameliorates DOX impaired aconitase activity and ATP levels and also improved cellular respiration. (Figure 6A) Our results indicate that PAESe improved aconitase activity that was altered by DOX [t (6) = 5.483; *p* = 00.15] and (t [6) = 4.279; *p* = 0.0052]. We observed a dose-dependent reduction in the ATP in our H9C2 cardiomyoblasts after DOX treatment. However, PAESe prevented the DOX-induced reduction in ATP levels in H9C2 cardiomyoblast cells (Figure 6B), (t (10) = 4.988, *p* = 0.0005 (5 µM), (t (10) = 3.413; *p* = 0.0066 (10 µM), (t (10) = 2.735, *p* = 0.0210 (20 µM), (t (10) = 4.032, *p* = 0.0024 (50 µM). We also observed that Dox (10 µm for 24 h) significantly impaired cellular respiration (Figure 6C) [basal (t (4) = 6.1155; *p* = 0.0036)] and [uncoupled (t (4) = 4.720; *p* = 0.0092)]. Furthermore, we also observed that PAESe conferred protection against DOX-mediated reduction in reduction in basal oxygen consumption rates [basal (t (4) = 3.089; *p* = 0.0366)] and [uncoupled (t (4) = 3.358;*p* = 0.0284)]. These findings are in line with our previously published work, where we observed similarly reduced oxygen consumption rates in FRDA fibroblasts and cells with FXN knockdown. Our working model (Figure 7A), describes together how PAESe regenerates glutathione and protects Frataxin from damage associated from doxorubicin.

## Discussion

Cardiotoxicity associated with anthracycline therapy develops as a progressive form of cardiomyopathy after the termination of the dosage regimen. Previously, we have demonstrated that administration of DOX (5 mg/kg) to athymic mice resulted in significant toxicity, reducing the median survival rates mice by 50% after 8 weeks of dosing (Kang et al., 2014). Acute dosing in the clinical setting induces the development of ventricular conduction abnormalities, including ST and T wave abnormalities, prolonged QT interval, and ventricular arrhythmias (Rudzinski et al., 2007). However, anthracycline dosing regimens are regarded as chronic due to their cumulative and dose-dependent impact on the heart over time. Further, the onset of cardiomyopathy from chronic dosing appears only after cessation of anthracycline therapy (Grenier and Lipshultz, 1998; Lipshultz et al., 2008; Fadol and Lech, 2011; Liang et al., 2020). However, the mechanisms associated with the development of DOX-mediated cardiotoxicity are unclear and complicated.

To better understand the mechanisms that are involved in the development of cardiotoxicity, we determined parameters of cardiac function after cumulative dosing of 25 mg/kg of DOX. Our dosing regimen is analogous to other studies that investigate the effects of similar chronic dosing on the myocardial architecture. As determined by our MRI analysis, we observed enlargement in the anterolateral and lateral segments of the left ventricle and reduced hemodynamic performance suggesting a compensatory state that potentially can progress to heart failure over time. To further explain the consequent cardiac related pathological condition of Dox treatment in our mice, we observed elevated cardiac troponin levels in circulation, thus suggesting cardiotoxicity. These results are in line with other reports in DOX treated athymic mice, where similar cumulative dosing regimen (25 mg/kg) induced an increase in cTnI levels (Sonowal et al., 2017). These findings helps to explain the reduced ejection volumes and reduced cardiac outputs in our mice.

The effects of DOX on the development of cardiac hypertrophy at the subcellular level can be studied in parallel with the impact on the dysregulation mitochondrial respiration, membrane potential and ROS levels. Mitochondrial damage has been reported to be a major target for DOX-mediated cardiomyopathy (Jarreta et al., 2000; Lev et al., 2004). DOX induces free oxygen radical production in the heart, which is a contributing factor for the disruption of mitochondrial bioenergetics (Keizer et al., 1990; Cadenas and Davies, 2000; Carvalho et al., 2014). However, alternative mechanisms associated with DOX cardiotoxicity may also contribute to the dysregulation of mitochondrial dynamics. Therefore, identifying the molecular and cellular targets that are altered by DOX in the mitochondria will help develop novel therapeutics to circumvent the adverse cardiogenic effects of DOX in cancer patients. The present study elucidated a potential mechanism by which DOX exposure induces a compensatory hypertrophic cardiac hypertrophy by implicating FXN as a crucial mediator in this process. Our previous reports have observed that reduced FXN levels induce an increase in mitochondrial iron accumulation and the subsequent ROS formation and H9C2 cell enlargement. Further, that by chelating this iron, using pyridoxal isonicotinol hydrazine (PIH) the DOX mediated H9C2 cardiomyocyte enlargement was significantly reduced (Mouli et al., 2015).

PAESe is a phenylaminoalkyl selenide is a strong antioxidant that scavenges reactive oxygen species while also serving as an antihypertensive activity agent (May et al., 1987; May et al., 1997; May and Pollock, 1998). Furthermore, PAESe mechanistically serves as a selenoxide product that is recycled back to its selenide form by cellular reductants including ascorbate and glutathione (May et al., 1987; May et al., 1997; May and Pollock, 1998). In addition, we have observed that PAESe is stable *in vitro*, displays very low toxicity *in vivo* (rats and mice), and displays no complicating side effects. Recently, we demonstrated that PAESe exhibits cardio-protective effects against anthracyclin-mediated cardiotoxicity *in vitro* cell culture as well as *in vivo* antitumor activity (Kang et al., 2011). However, the exact mechanism (s) is not known; furthermore, mitochondrial damage exists as an underlying factor in the progression of cardiomyopathy to heart failure (Koutnikova et al., 1998; Jarreta et al., 2000; Lev et al., 2004; Rudzinski et al., 2007; Schmitz et al., 2012; Bayeva et al., 2013). Clinical findings support the notion that human patients with FRDA display reduced FXN levels, develop life-threatening hypertrophic cardiomyopathy (Jauslin et al., 2002). In close agreement with this, our *in vivo* and *in vitro* models with DOX have demonstrated a significant reduction in FXN expression (Mouli et al., 2015). Furthermore, that PAESe prevents DOX mediated cardiac hypertrophy via reducing the increase in mitochondrial free iron accumulation (Kang et al., 2015). However, our findings indicate that progressive damage to FXN induced by DOX was central in mediating mitochondrial iron overload. Our hypothesis was confirmed by our previous findings in FXN-KD cardiomyocyte cell lines, which displayed a similar increase in mitochondrial iron, as found in DOX treated cardiomyocytes (Mouli et al., 2015). However, our FXN-OE cardiomyocytes demonstrated stable iron homeostasis in the mitochondria in the presence of DOX and prevented the deleterious effects of DOX on the mitochondrial iron utilization pathways. Furthermore, we previously observed insignificant amounts of ROS production in FXN-OE cell lines after DOX treatment (Mouli et al., 2015). Importantly, our previously published study linked increased mitochondrial iron levels following DOX treatment to increased ROS production that resulted in mitochondrial injury, including reduced mitochondrial membrane potential (ΔΨ_m_) (Mouli et al., 2015). Conversely, the use of the iron chelator, PIH was observed to help maintain mitochondrial ΔΨ_m_ in H9C2 cells treated with DOX. Thus confirming that mitochondrial iron overload with the formation of mitochondrial ROS. Maintenance of mitochondrial ΔΨ_m_ in cardiomyocytes is vital to maintain the high energy requirements for viability and function and attenuation of ΔΨ_m_ induces injury to cardiomyocytes Gladyshev et al., 1996. We assessed ΔΨ_m_ in cardiomyoblasts using the fluorescent probe TMRE. Interestingly, FXN-OE cells treated with DOX were protected from the adverse impact of DOX on ΔΨ_m_. In the present study, we observed that PAESe preserved the normal (lower) mitochondrial ΔΨ_m_, (Figures 5D,E). However, our study had some deficits, including our animal model. We chose to use an athymic mouse model for our studies. This model lacks the T-cell immunity and thus are immunocompromized in many ways. In this manner, our model investigated the direct impact of DOX on the cardiomyocytes. Our goals were to determine the mechanism by which PAESe protects the heart against DOX mediated cardiotoxicity. We acknowledge that the immune system also has a major impact on the inflammation, hypertrophy and development of compensatory myocardial damage to DOX. Future work will investigate whether PAESe protects from DOX mediated cardio toxicity in a model, where the impact of the full immune system is involved, including potential T cell mediated immune damage to the heart.

Our data suggest that DOX induces an increase in mitochondrial ROS formation resulting in early ubiquitination of FXN protein by activating E3 ligase at the current DOX as inferred by other studies demonstrating mechanisms for FXN turnover in FRDA patients (Landré et al., 2014; Rufini et al., 2014). These hypotheses will be explored in detail in our future studies. Reports from FRDA models, have demonstrated severe mitochondrial energy deficits as demonstrated by reduced oxidative capacity due to depleted FXN levels and impaired Fe-S cluster enzyme activities, diminished respiratory function, and reduced ATP levels (Thierbach et al., 2005; González-Cabo and Palau, 2013). Similar events were observed in our DOX treated cardiomyocytes, which displayed a severe reduction in the activities of iron-sulfur containing enzyme aconitase, as well as ATP production and oxygen consumption levels. Further, we observed that by reducing the deleterious effects of DOX on FXN by PAESe, we preserve mitochondrial bioenergetics. Under stress, glutathione reductase maintains the physiological GSH/GSSG balance in an NADPH dependent fashion (Lushchak, 2012). Earlier reports have demonstrated that FXN deficient and FRDA cells can be affiliated with the deficiency of glutathione pool (GSH/GSSG), which serves as a free radical scavenger and co-factor for various mitochondrial antioxidants (Jauslin et al., 2002; Auchère et al., 2008). In line with these findings, we observed that PAESe prevented the DOX-mediated reduction of glutathione peroxidase enzyme activity (a requirement for glutathione replenishment). Previously we have confirmed that PAESe replenishes glutathione levels and FADH2 levels (Mouli et al., 2015).

Mechanistically, PAESe is oxidized to its selenoxide PAESe (O) form via dopamine-β-monooxygenase. Reduced ascorbate reduces the oxidized PAESe back to the reduced form resulting in the generation of oxidized ascorbate in the process. Ascorbate is then capable of reacting with PAESe and hydrogen peroxide to reduce Fe^3+^ back to Fe^2+^, resulting in lowering reactive oxygen species associated with iron. This source of ROS was confirmed in our previously published reports where the iron chelator pyridoxal isonicotinoyl hydrazine (PIH), was shown to reduce mitochondrial free iron levels and mitochondrial ROS levels. We have observed that catalytic regeneration of PAESe is also enhanced via Glutathione Peroxidase (GPx) reduction with glutathione (GSH) yielding glutathione disulfide (GSSG) (Figure 7). In addition, regeneration of PAESe enables mitochondrial ROS formation to be ameliorated at a much faster rate compared to normal physiological conditions, allowing for potent free radical scavenging and enhanced mitochondrial function. To implicate FXN in the signaling mechanism, as previously reported, our FXN-overexpressing (OE) cardiomyoblasts were protected from the unfavorable effects of DOX in the mitochondria and may be due to elevated anti-oxidative defenses (Mouli et al., 2015). Therefore these findings, taken together, suggest that PAESe offered protection against DOX-mediated adverse effects by significantly improving the mitochondrial energy flux and anti-oxidative capacity, thereby preserving the mitochondrial bioenergetics.

Our H9C2 cardiomyocytes treated with DOX displayed extensive ROS, which was prevented by PAESe. We have previously shown that the consequence of DOX on reducing FXN expression is to induce mitochondrial iron overload and thus enhance mitochondrial ROS formation via the Fenton reaction. Previously we confirmed these findings with the utilization of a mitochondria specific iron chelator, PIH. The consequences of these findings in PAESe treated cells on mitochondrial activity resulted in the preservation of the mitochondrial membrane potential against DOX insult. Lastly, our PAESe in a similar manner to FXN-OE cardiomyocytes (Mouli et al., 2015) demonstrated reduced cardiac hypertrophy in response to DOX treatment. These findings indicate that PAESe protected against DOX-induced iron overload, thus indicating a potential therapeutic mechanism towards mitigating the development of DOX mediated cardiac hypertrophy, as seen by our MRI data.

In summary, our findings indicate that FXN plays a significant role in the development of DOX mediated myocardial iron overload and that PAESe protects FXN from the impaired mitochondrial bioenergetics leading to progression of de-compensatory cardiomyopathy. Further that PAESe was found to be protective against DOX mediated mitochondrial dysfunction and the ensuing cardiac hypertrophy. Our future work will focus on determining how PAESe can prevent myocardial iron accumulation in chronic usage of DOX administration at extended time points following completion of DOX therapy. Second, we are currently developing the FXN overexpressing mouse model to understand better and justify the role of FXN in the DOX mediated damage to the mitochondria. Our FXN overexpressing mouse model will further help decipher the role of FXN in the development of DOX mediated cardiomyopathy.

## Data Availability

The raw data supporting the conclusions of this article will be made available by the authors, without undue reservation.
